# The polynucleotide kinase 3′-phosphatase gene (*PNKP*) is involved in Charcot-Marie-Tooth disease (CMT2B2) previously related to *MED25*

**DOI:** 10.1007/s10048-018-0555-7

**Published:** 2018-07-24

**Authors:** Alejandro Leal, Sixto Bogantes-Ledezma, Arif B. Ekici, Steffen Uebe, Christian T. Thiel, Heinrich Sticht, Martin Berghoff, Corinna Berghoff, Bernal Morera, Michael Meisterernst, André Reis

**Affiliations:** 1 Section of Genetics and Biotechnology, School of Biology, Universidad de Costa Rica, Sede Montes de Oca, San José, 2060 Costa Rica; 2 Neuroscience Research Center, Universidad de Costa Rica, San José, Costa Rica; 3 Neurology Department, Hospital San Juan de Dios, San José, Costa Rica; 4 Institute of Human Genetics, Friedrich-Alexander-Universität Erlangen-Nürnberg (FAU), Erlangen, Germany; 5 Institute of Biochemistry, Friedrich-Alexander-Universtät Erlangen-Nürnberg, Erlangen, Germany; 6 Klinikum der Justus-Liebig-Universität, Gießen, Germany; 7 Practice of Neurology, Gießen, Germany; 8 School of Biological Sciences, Universidad Nacional, Heredia, Costa Rica; 9 Institute of Molecular Tumor Biology, Wilhelms Universität, Münster, Germany

**Keywords:** CMT, CMT2B2, AOA4, PNKP, MED25

## Abstract

Charcot-Marie-Tooth disease (CMT) represents a heterogeneous group of hereditary peripheral neuropathies. We previously reported a CMT locus on chromosome 19q13.3 segregating with the disease in a large Costa Rican family with axonal neuropathy and autosomal recessive pattern of inheritance (CMT2B2). We proposed a homozygous missense variant in the Mediator complex 25 (*MED25*) gene as causative of the disease. Nevertheless, the fact that no other CMT individuals with *MED25* variants were reported to date led us to reevaluate the original family. Using exome sequencing, we now identified a homozygous nonsense variant (p.Gln517ter) in the last exon of an adjacent gene, the polynucleotide kinase 3′-phosphatase (PNKP) gene. It encodes a DNA repair protein recently associated with recessive ataxia with oculomotor apraxia type 4 (AOA4) and microcephaly, seizures, and developmental delay (MCSZ). Subsequently, five unrelated Costa Rican CMT2 subjects initially identified as being heterozygous for the same *MED25* variant were found to be also compound heterozygote for *PNKP*. All were heterozygous for the same variant found homozygous in the large family and a second one previously associated with ataxia (p.Thr408del). Detailed clinical reassessment of the initial family and the new individuals revealed in all an adult-onset slowly progressive CMT2 associated with signs of cerebellar dysfunction such as slurred speech and oculomotor involvement, but neither microcephaly, seizures, nor developmental delay. We propose that *PKNP* variants are the major causative variant for the CMT2 phenotype in these individuals and that the milder clinical manifestation is due to an allelic effect.

## Introduction

In 2001, we reported mapping of a locus for an axonal Charcot-Marie-Tooth disease with autosomal recessive pattern of inheritance to chromosome 19q13.3 (CMT2B2, MIM 605589). Affected members of a Costa Rican family (CR-P) presented with a symmetric motor and sensory neuropathy, distal muscle wasting, impaired deep tendon reflexes, and age at onset between 28 and 42 years. Electrophysiological studies revealed an axonal degenerative process (CMT2) [1]. Fine-mapping allowed refining of the critical interval to 1 Mb encompassing a total of 53 established or putative genes. An exhaustive search for the causative variant with Sanger sequencing of all exons identified a single missense variant (c.1004C>T) p.Ala335Val in the Mediator complex 25 (*MED25*) gene, encoding a subunit of an RNA polymerase II transcriptional regulator complex. The variant is located in a proline-rich region with high affinity for SH3 domains of the Abelson type, and we could demonstrate that it leads to a decreased binding specificity. Also, we showed that in mice and rats *Med25* is coordinately expressed with *Pmp22* gene, a gene involved in the Charcot-Marie-Tooth disease [2]. More recently, an individual was identified with an axonal form of CMT carrying compound heterozygous variants (p.Ala335Val and p.Pro656Thr) in *MED25*, although this individual also presented with additional variants in other CMT-related genes [3]. Finally, suppression of *med25* in Zebrafish caused damage in the axon of peripheral neurons [3]. All these findings suggested a causative role for MED25 in CMT2.

Despite this, it was remarkable that until now no additional CMT families with variants in *MED25* were identified. To exclude that other variants were missed in the initial Sanger-based analysis and given that meanwhile, high-throughput sequencing became available, we decided to perform exome sequencing in several affected members of the CR-P family. This led now to the identification of homozygous nonsense variant in the last exon of an adjacent gene encoding the polynucleotide kinase 3′-phosphatase (*PNKP*). Although this variant was already identified in our original screen, it was nevertheless discarded as the *PNKP* gene annotation used at the time included an upstream stop codon causing the variant to be mapped outside the coding sequence.

PKNP catalyzes the 5-prime phosphorylation of nucleic acids and also has an associated 3-prime phosphatase activity, an important function in DNA repair following ionizing radiation or oxidative damage [4]. It acts as a 5′-kinase/3′-phosphatase to create 5′-phosphate/3′-hydroxyl termini, which are a necessary prerequisite for ligation during repair [5]. Homozygous variants in this gene were previously linked to progressive cerebellar atrophy and polyneuropathy [6] and recently, to both severe ataxia with oculomotor apraxia 4 (AOA4, MIM 616267) or microcephaly with seizures and developmental delay (MCSZ, MIM 613402) [7]. Our findings imply that this DNA repair enzyme is involved in a broader phenotype, including a mild axonal peripheral polyneuropathy.

## Methods

### Variant detection

**Fig. 1:**
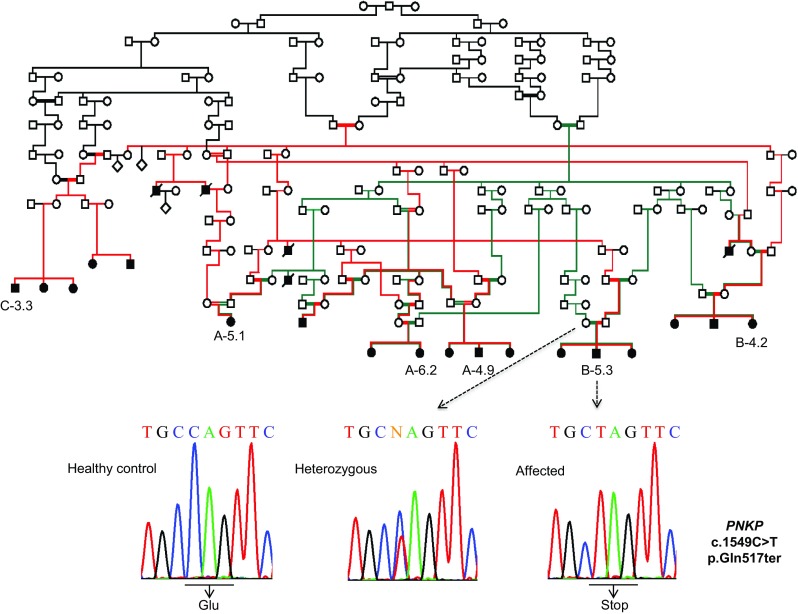
Pedigree of the CMT-P family and electropherograms showing the PNKP c.1549C>T variant. All members of the original family affected with CMT2B2 are homozygous for the mutant allele leading to a c-terminal nonsense mutation at codon 517

We used DNA samples from 41 family members of the original CMT2B2 Costa Rican family, including 18 affected persons. Individuals A4.9, A5.1, A6.2, B5.3, and C3.3 [1] were selected for exome sequencing (Fig. 1). Automated library preparation was performed with a liquid handling robot and an appropriate library prep kit (both Beckman-Coulter) and enriched for the exome using Agilent SureSelect human all exon v6. Samples were then sequenced in 2 × 125 bp paired-end runs on a HiSeq2500 system with the Illumina SBS kit v4. Subsequent mapping of reads was performed using the bwa-MEM software version 0.7.8, followed by deduplication with Picard 1.111, and local realignment using GATK 3.1.1. To identify variants from refseq-hg19, calling was performed with GATK HaplotypeCaller, GATK-UnifiedGenotyper, Platypus, freeBayes, and SNVer, respectively. Subsequent annotation was accomplished with Annovar.

### Filtering steps

Variant analysis considering a homozygous recessive mode of inheritance was performed using the Next Generation Sequencing Variant Analyzer (NGS-VA), an in-house developed tool. To minimize the number of false positives, we included only variants which were covered by at least 10% of the average coverage of the patient’s exome. We excluded all variants with a frequency > 0.001 in the 1000 Genomes Project, the Exome Variant Server and the Exome Aggregation Consortium server. Furthermore, we restricted our analysis to exonic variants and variants in canonical splice sites ranging from − 12 to + 5. As we considered a stronger impact of protein-altering variants, we further excluded synonymous variants from our analysis. The remaining variants were manually examined with the Integrative Genomics Viewer to exclude artifacts. Taking into account that the Costa Rican family shows consanguinity and the same haplotype is linked to the CMT locus, the NGS-VA selected only variants present in all affected members tested. The exome analysis was performed according to Hauer et al. (2017) [8].

### Validation

The identified *PKNP* variant was validated by Sanger sequencing using flanking primers: PNKP-50364522-F (5′-ATGTCTAAAGTGCTCATGCCAGG-3′) and PNKP-50364522-R (5′-GGTACTGTTGGGGATAGCAGG-3′). DNA samples from all family members were used to confirm segregation. PCR conditions are available on request. Bidirectional direct sequencing was implemented using the BigDye Terminator Cycle Sequencing Kit (Applied Biosystems) on a 3730 capillary sequencer (Applied Biosystems). Sequence traces were evaluated using the DNAStar software package.

Other five CMT2 individuals previously identified to be heterozygous for the *MED25* variant p.A335V, donated DNA samples and were likewise analyzed for the p.Q517X or other *PNKP* and *MED25* variants (primers and conditions are available on request). Also, exome sequencing was performed for one of these additionally recruited individuals (CMT1101).

Informed consent was obtained from all participants included in the study. The study was approved by the institutional review board at University of Costa Rica.

### Protein modeling

The structure of human PNKP was modeled based on the crystal structure of murine PNKP (PDB code: 3ZVN) [9] exhibiting 92% sequence similarity. Rasmol [10] was used for structure analysis and visualization.

### Clinical analysis

All affected individuals from the original large family underwent clinical and electrophysiological evaluations [2]. Some of them are still alive, and we were able to reassess two of them (P009 and P178). Also, the five patients of the five additional families were clinically and electrophysiologically studied some years ago, but two of them could be likewise reanalyzed (CMT1003 and CMT1190). Clinical reevaluation of the four reanalyzed patients of both the large and other families was performed by a single neurologist (SB-L). Standard clinical, electrophysiological examinations were recorded by ALPINE BIOMED Keypoint Portable EMG Unit by the same physician. To evaluate if patients presented with brain alterations, four affected individuals were scanned with a 1.5-T MRI using T1-weighted and T2-weighted both axial and coronal, FLAIR, and diffusion-weighted sequences.

## Results

### Exome sequencing

Seventy-two homozygous variants were identified in the five exome-sequenced individuals of the original Costa Rican family with CMT2B2, but only one was shared by all five. In this way, a transition c.C1549T in exon 17 of the polynucleotide kinase 3′-phosphatase (*PNKP*) gene (rs774995635) was found to be homozygous for all affected members of the family (Fig. 1). This variant has a frequency of 18/245,644 (gnomAD database All) and causes a nonsense mutation (p.Gln517ter) predicted to truncate the last five amino acids. *PNKP* is located within the critical linkage interval on chromosome 19q13.33 previously described for this family [2].

When using more relaxed allele frequency parameters to admit variants with a general frequency < 0.005%, the variant p.Ala335Val in *MED25* could also be detected. Segregation analysis of the *MED25* and *PNKP* variants demonstrated that they fully cosegregate indicating that they are present on the same haplotype in all affected individuals. Interestingly, all the unrelated individuals heterozygous for the *MED25* variant not belonging to the extended family were also heterozygous for this *PNKP* variant, suggesting that this is an ancestral founder haplotype.

**Fig. 2:**
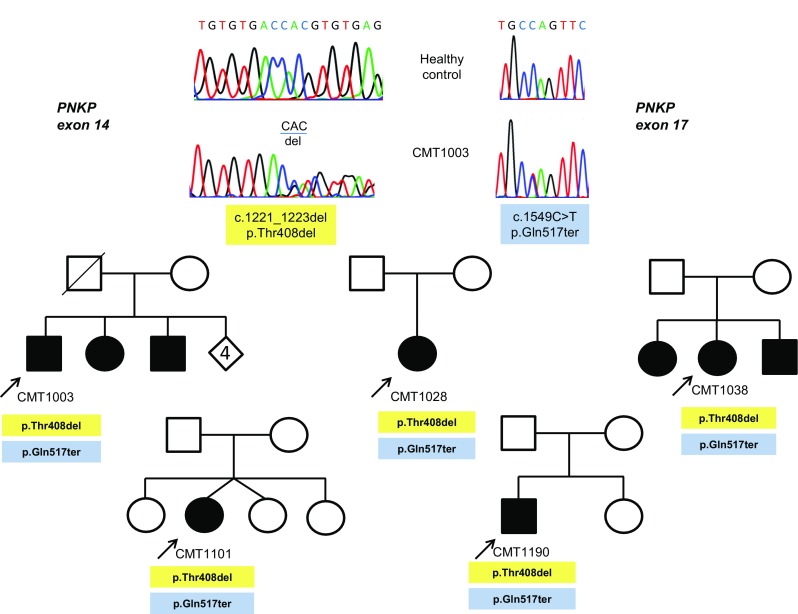
Pedigrees and electropherograms of five additional Costa Rican CMT families with affected members due to compound heterozygosity for two PNKP variants, the mutated alleles c.1549C>T, found in the large initial family, and the c.1221_1223del, previously related to ataxia with oculomotor apraxia

Analysis of the entire *PNKP* coding sequence in the five Costa Rican CMT2 patients not belonging to the extended family but also heterozygous for *MED25* variant p.Ala335Val identified the same additional *PKNP* variant in all five of them. It consists of a three-base deletion in exon 14, c.1221_1223del, previously associated with autosomal recessive Ataxia-Oculomotor Apraxia 4 (AOA4; MIM 616267, variant .0007). This variant results in the deletion of residue Thr408 (Thr408del) (Fig. 2) and was observed in the gnomAD database (rs770849181) with a general frequency of 15/214338. Through segregation analysis, it was observed that these individuals are compound heterozygous for the *PNKP* variants identified (c.1221_1223del/c.C1549T). No additional MED25 variants were found in any of these individuals.

**Fig. 3:**
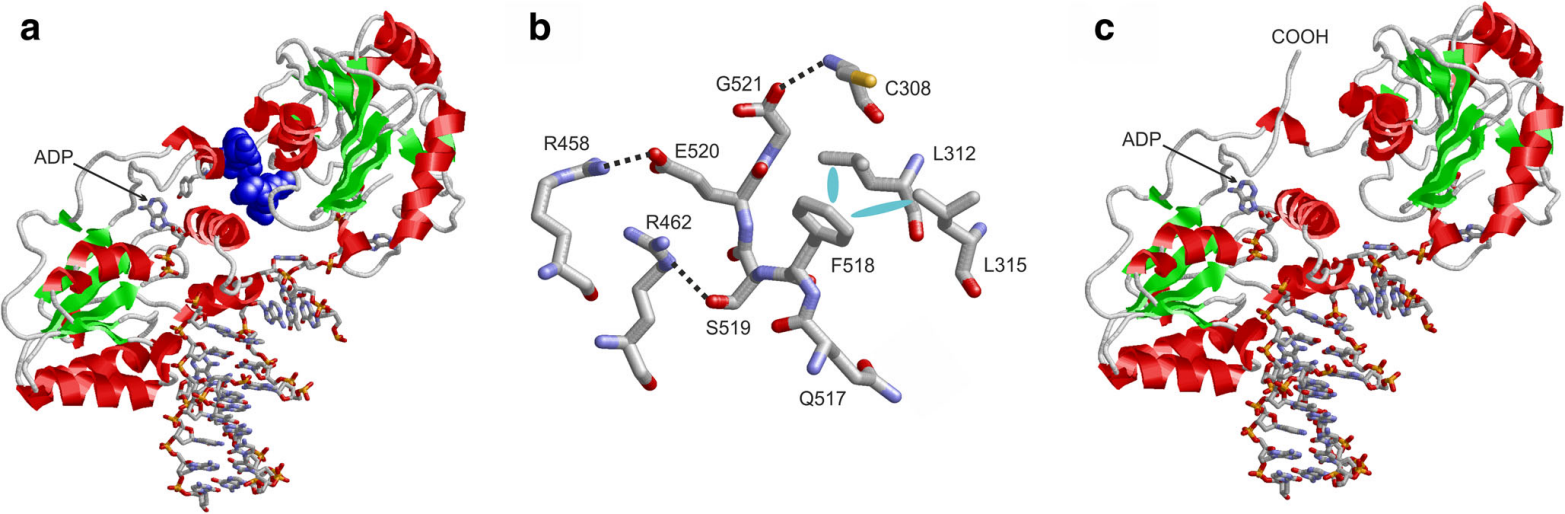
Effect of the Gln517ter variant on PNKP structure. **a** Structure of DNA-bound wildtype PNKP. The protein is shown as ribbon and residues 517–521, which are lacking in the variant, are shown in the blue space-filled presentation. DNA, ADP, and residue Y515 as part of the ADP-pocket are shown in stick presentation. **b** Detailed view showing the interactions of residues 517–521 of wildtype PNKP. Polar and hydrophobic interactions are indicated by black dotted lines and cyan ellipses, respectively. **c** Structure of Gln517ter PNKP schematically depicting the rearrangement of the shorter COOH-terminus due to the lack of interaction with the remaining domains

PNKP contains a kinase and a phosphate domain that are both involved in DNA binding. The C-terminus of the protein is buried between these two domains (Fig. 3a). Wildtype residues Q517-G521, which are lacking in the p.Gln517ter variant, form extensive interactions with residues from the phosphatase domain (C308, L312, L315) and the kinase domain (R458, R462) (Fig. 3b).

### Clinical investigation

All patients from the large Costa Rican family and the five additional families were clinically and electrophysiologically diagnosed with axonal peripheral polyneuropathy. On general examination (Table 1), the four reevaluated individuals presented with mild lumbar scoliosis and a claw hand, but only the youngest male (CMT1190, compound heterozygous) had pes cavus and hammertoes. The neurological examination did not show evidence of cognitive impairment, microcephaly [11], or seizures. None of them were affected by movement disorders, but all had slurred speech, most pronounced in the oldest male (CMT1003, compound heterozygous).

**Table 1:** Clinical signs and imaging features of CMT2B2 individuals carrying PNKP mutations

	CMT1003	CMT1190	B4.2	A6.2
Gender	Male	Male	Female	Female
Age	55	26	59	55
Decade of age at onset	Third	Third	Third	Third
First sign	Polyneuropathy	Polyneuropathy	Polyneuropathy	Polyneuropathy
More prominent sign	Polyneuropathy	Polyneuropathy	Polyneuropathy	Polyneuropathy
OMA	+++	+	–	–
Slurred speech	+++	+	+	+
Mobility	5 years in wheelchair	Mobile, mild gait ataxia	Intermittent mobile	Intermittent mobile
Dystonia	–	–	–	–
Cognitive impairment	–	–	–	–
UE muscle strength (I/D)	3/0	4/3	3/0	3/0
LE muscle strength (P/I/D)	4/0/0	5/3/0	5/0/0	5/0/0
Reflexes (UE/knee/ankle)	1/0/0	2/0/0	1/0/0	1/0/0
UE sensory involvement (T/Pa/V/Po)	2/1/0/0	2/1/0/0	2/1/0/0	2/1/0/0
LE sensory involvement (T/Pa/V/Po)	0/0/0/0	1/0/0/0	0/0/0/0	0/0/0/0
Atrophy	Intrinsic muscles of hands and feet, calves	Intrinsic muscles of hands and feet, calves	Intrinsic muscles of hands and feet, calves	Intrinsic muscles of hands and feet, lymphedema
Deformities	Claw hand	Claw hand, pes cavus and hammertoes	Claw hand	Claw hand
Pyramidal signs	–	–	–	–
Obesity	–	–	–	+
MRI findings	CA, no WM abnormalities	CA, no WM abnormalities	CA, no WM abnormalities	CA, no WM abnormalities
Head circumference (centile against height and gender) (21)	55.8 cm (25th)	58.3 cm (75th)	53 cm (50th)	52.5 cm (25th)

*OMA* oculomotor apraxia, *UE* upper extremity, *LE* lower extremity, *P* proximal (knee extensor or flexor), *I* intermediate (hand extensor or flexor (UE), foot extensor or flexor (LE), *D* distal (intrinsic hand muscles (UE), intrinsic foot muscles (LE)). Motor scale: *5* normal; *4* mild weakness; *3* ability to lift against gravity; *2* not able to lift against gravity, but movement visible; *1* no movement, but tendon contraction visible; *0* complete paralysis. Reflexes/sense of vibration or position: *2* normal, *1* reduced, *0* absent, *T* touch, *Pa* pain, *V* vibration, *Po* position. Sensory involvement: *2* normal; *1* mildly reduced, distally to wrist level (*UE*) or malleoli level (*LE*); 0 severely reduced, distally to elbow level (*UE*) or knee level (*LE*). *CA* cerebellar atrophy. *WM* white matter

The ocular movement exploration revealed that the oldest male was affected by a complete inability to start voluntary eye movements, either in the vertical or horizontal planes. However, he exhibited vestibulo-ocular reflex (VOR) and was able to keep the gaze fixed at a point while performing passive movements of the head. The youngest male (CMT1190) presented with slow saccades and difficulty in initiating voluntary changes in gaze with preserved VOR. Among the affected females (A6.2 and B4.2) homozygous for the p.Gln517ter variant in *PNKP*, only the older one had mild difficulty in initiating saccades but without oculomotor apraxia, while the younger presented with no apparent ocular movement problems.

The motor strength in the proximal upper limbs was entirely preserved, but palmar grip strength was moderately reduced. In the older patients, proximal lower limb strength was moderately reduced, but in the youngest individual (CMT1190) it was completely preserved. Distally, they all were weak in all limbs, muscle atrophy of the calves and the intrinsic muscles of the hand and foot was observed; the first muscle group could not be assessed in the youngest woman (A6.2) because she presented with lymphedema.

Sensory examination demonstrated marked decrease of vibration and position sensations in either upper or lower extremities of all patients; touch and pain sensations were preserved only in the upper extremities. Deep-tendon reflexes were absent in the lower extremities. The youngest male (CMT1190) had a normal finger to nose test, both females (A6.2 and B4.2) showed severe slowness of motor execution in the finger to nose test, and the older man (CMT1003) was unable to perform the test. The youngest male was able to walk without support but had a wide-based gait. The oldest woman could walk short distances with a four-point walking device. Finally, the oldest male was wheelchair-bound for 5 years and was dependent for daily living activities.

**Fig. 4:**
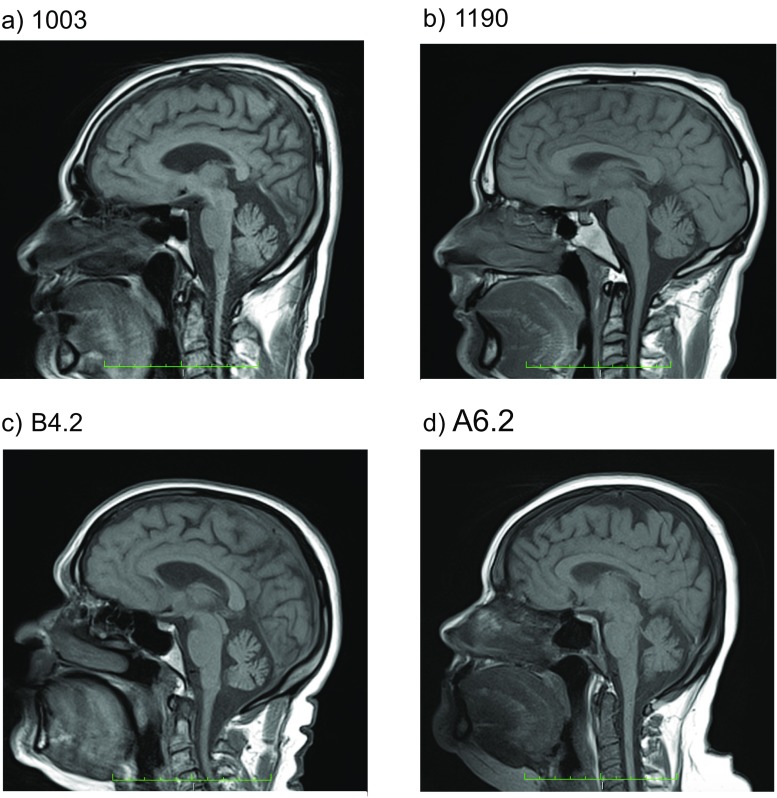
Midsagittal T1-weighted brain MRI of four CMT2B2 individuals showing mild cerebellar atrophy. **a** CMT1003. **b** CMT1190. **c** B4.2 (**C**). **d** A6.2

**Fig. 5:**
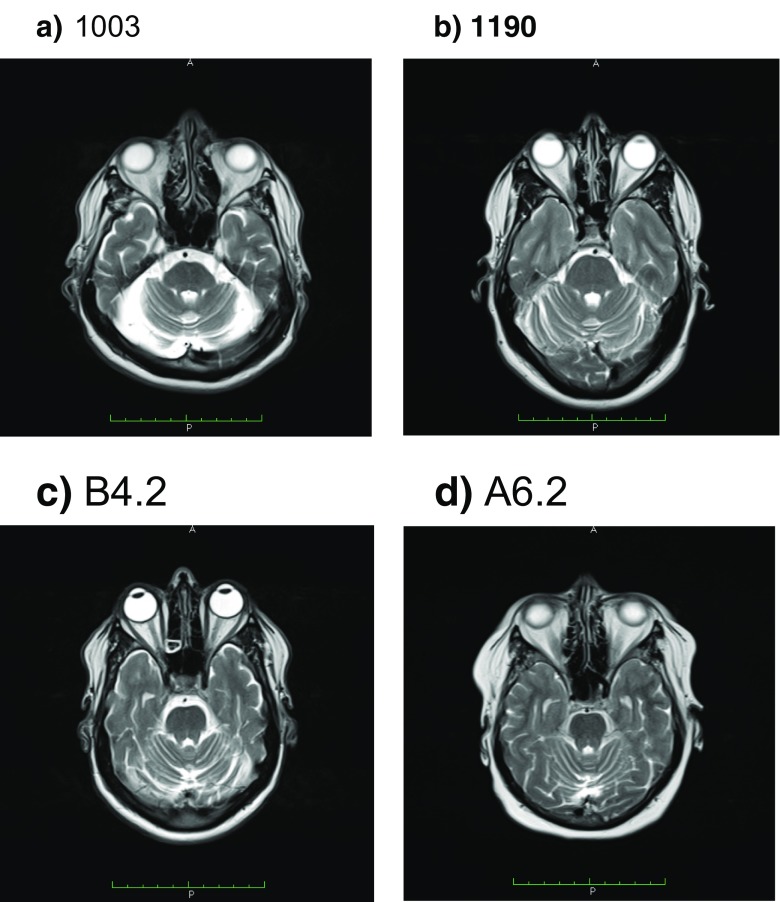
Axial T2-weighted brain MRI of the four individuals showing mild cerebellar atrophy. **a** CMT1003. **b** CMT1190. **c** B4.2. **d** A6.2

**Fig. 6:**
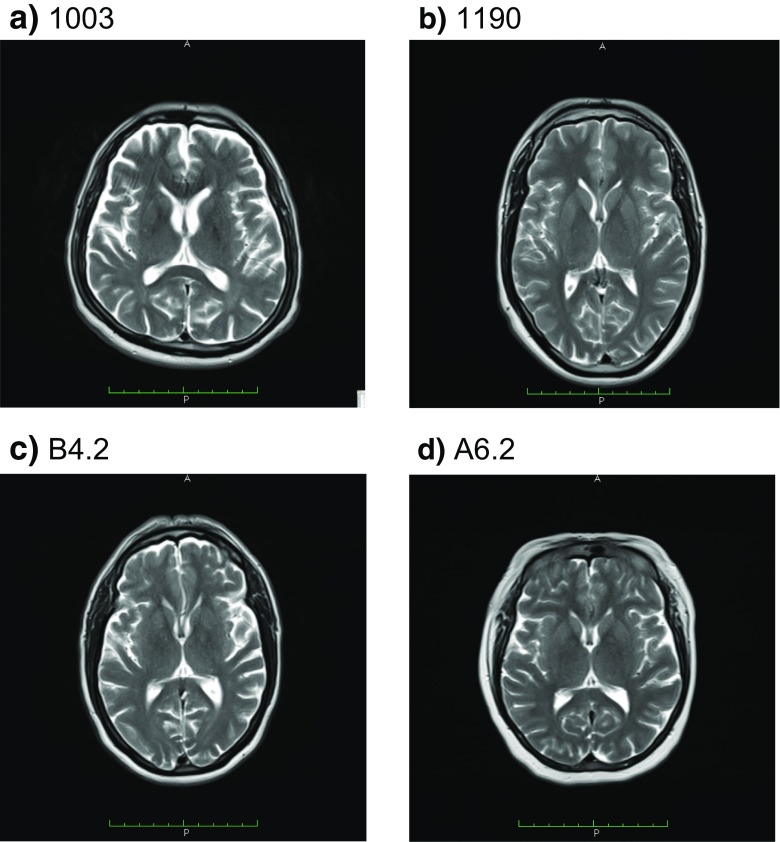
Axial T2-weighted brain MRI of the four individuals at a ganglionar level shows no brain structures lesions. **a** CMT1003. **b** CMT1190. **c** B4.2. **d** A6.2

Electrophysiological studies (Table 2) showed a severe axonal sensory-motor polyneuropathy of the four limbs. In the three patients who maintain mobility, the electrophysiological analysis demonstrated a severe reduction of the CMAP of affected nerves with relative preservation of F-waves latency and secondary slowing of the MNCV in the elder individuals. Meanwhile, in the wheelchair-bound individual, the CMAP could not be measured. MRI of the brain revealed cerebellar atrophy with no white matter abnormalities, brain atrophy, nor brainstem atrophy, in all studied individuals (Figs. 4, 5, and 6). Laboratory findings (Table 3) showed albumin levels practically normal, with mild elevation of cholesterol levels in two of them, and elevation between 1.1 and 1.5 times of the alpha-fetoprotein in all but the youngest patient. Just two patients had a mild elevation of IgE levels.

**Table 2:** Electrophysiological data of patients carrying PNKP mutations

Patient	Nerve	Motor NCS				Sensory NCS	
		DML (ms) right/left	MNCV (m/s) right/left	F-wave latencies (ms) right/left	Distal CMAP (mV) right/left	SNCV (m/s) right/left	SNAP (uV) right/left
CMT1003	Median	−/−	−/−	−/−	−/−	−/−	−/−
	Ulnar	−/−	−/−	−/−	−/−	−/−	−/−
	Tibial	−/−	−/−	−/−	−/−		
	Peroneal	−/−	−/−	−/−	−/−		
CMT1190	Median	4.1/4.4	44.6/38.0	28.3/31.2	11.9/4.7	−/−	−/−
	Ulnar	3.4/4.2	39.5/40.2	22.0/21.9	4.0/3.8	−/−	−/−
	Tibial	−/−	−/−	−/−	−/−		
	Peroneal	0.0/−	−/−	−/−	0.0/−		
B4.2	Median	9.8/−	17.8/−	−/21.0	0.0/−	−/−	−/−
	Ulnar	7.7/−	−/−	−/20.8	0.0/−	−/−	−/−
	Tibial	−/−	−/−	−/−	−/−		
	Peroneal	−/−	−/−	−/−	−/−		
A6.2	Median	6.1/3.9	31.5/41. 5	54.6/30.9	0.1/7.7	−/−	−/−
	Ulnar	4.2/4.1	35.0/39.3	53.5/50.4	0.2/0.8	−/−	−/−
	Tibial	−/−	−/−	−/−	−/−		
	Peroneal	−/−	−/−	−/−	−/−

**Table 3:** Laboratory findings of patients carrying PNKP mutations

Parameter	CMT1003	CMT1190	B4.2	A6.2	Reference
Albumin	2.13	3.24	3.45	3.51	3.5–4.8 g/dL
Cholesterol	144	274	244	159	< 200 mg/dL
HDL	36.5	40	36	52.5	> 35 mg/dL
LDL	64.7	186	135	85.6	< 130 mg/dL
Triglyceride	112	217	407	59.6	30–150 mg/dL
IgE	80.2	170.3	43.7	155	< 100 UI/mL
Alfa-fetoprotein	7.36	2.26	6.24	8.54	0.5–5.5 UI/mL

## Discussion

We provide evidence that a variant in the polynucleotide kinase 3′-phosphatase (PNKP) gene is responsible for the CMT2B2 in a large Costa Rican family (CR-P), previously associated with a variant in the subunit 25 of the Mediator complex (MED25) gene. Although all affected members of this family are homozygous both for the p.Ala335Val variant in MED25 and p.Gln517del in PNKP, we have identified five additional individuals from other Costa Rican CMT2 families, who are heterozygous for the MED25 variant (without additional MED25 variants) but compound heterozygous for two PNKP variants (p.Gln517ter/p.Thr408del). This indicates that variants on PNKP are the principal cause of the disease. PNKP is located in the original linkage interval and it was tested in our patients. Nevertheless, the c. C1549T variant was initially not considered, as the annotation of that gene was incomplete when we performed the first Sanger sequencing analysis in 2001. The variant is located in what is now recognized at PNKP’s last exon.

**Fig. 7:**
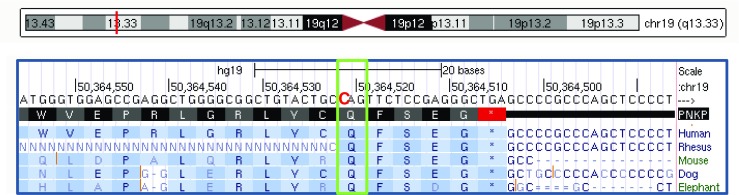
Homology analysis of the PNKP protein with the Gln517ter mutation highlighted with a green box. The last five amino acids of the enzyme (QFSEG) are highly evolutionary conserved in mammals

The PNKP variant p.Gln517ter is a rare variant with a frequency of 18/245,644 (gnomAD All), in agreement with what is expected for a deleterious mutation. Aminoacids at positions 517–521 of PNKP are very well evolutionary conserved in diverse species (Fig. 7) suggesting that C-terminal tail is critical for its function. The Gln517ter variant, homozygous for the affected individuals of the large family, causes the loss of those amino acids of the enzyme, that play a role in the stabilization of the protein, anchoring the kinase domain to the phosphatase domain [12]. Another mutation in PNKP at the same position (Gln517Leufs*24) has been shown to cause ataxia (including polyneuropathy) [12]. Protein modeling of PNKP with and without the Gln517ter mutation shows that this mutation is predicted to be pathogenic since the fixation of the C-terminus appears essential for stabilizing the relative domain orientation and for stabilizing the conformation of the ADP-binding site, which involves Y515 close to the site of truncation. Due to the lack of the stabilizing interactions with the other domains, the shorter carboxy-terminus of the variant is predicted to become flexible (Fig. 3c) thus leading to a distorted ADP-binding site and consequently a reduced enzymatic activity. This result in that damaged DNA, mainly by oxidative stress at the nervous system, cannot be repaired efficiently, with a subsequent transcription interference and ultimately cell death [13]. In the present study, we investigated the eldest individuals carrying mutations in PNKP to date. Contrary to what is described for AOA4 [12, 14–17], the four reassessed individuals developed normally until the beginning of the third decade of life when gait disturbances and falls began. The disease in our patients progresses slowly, requiring a wheelchair near the sixth decade. Proximal muscle strength is preserved even in patients with the most advanced disease, and deterioration in gait was associated with postural instability rather than weakness. Cerebellar involvement occurs in all individuals, including the youngest with slurred speech-language and a wide-based gait. The two reanalyzed compound heterozygous individuals presented with oculomotor apraxia, more severe in the oldest (CMT1003) than in the youngest (CMT1190). This sign was not present in the reexamined homozygous individuals. In fact, the only finding on them related to ocular motility is the presence of slow saccadic movements in the older woman (B4.2). However, a 57-year-old male (B4.1) sibling of female B4.2 also homozygous for the p.Gln517del variant, presented with severe oculomotor apraxia and is currently wheelchair-bound. Oculomotor apraxia was not observed in the CMT2B2 individuals investigated 20 years ago.

Laboratory findings showed a small coincidence in the pattern of the biological parameters as described in other individuals that carry PNKP mutations [12, 15–17]. Only patient CMT1190 presents hypoalbuminemia, hypercholesterolemia, and elevated IgE with normal alpha-fetoprotein as described in other patients (Table 4). The pattern of elevation or decrease of these biological parameters was not consistent in the other patients described in this study. Besides, there is no relationship between the severity of the phenotype and the levels of alpha-fetoprotein, IgE and albumin, contrary to what it has been reported in other patients with AOA4 [16, 17]. Curiously, our female patient that presents lower limb lymphedema, has no albumin alteration as described previously in a Norwegian female patient, undermining the theory that lymphedema was secondary to hypoalbuminemia [15].

**Table 4:** Comparison of laboratory findings of our patients with literature descriptions

Parameter	CMT1003	CMT1190	B4.2	A6.2	Bras et al.	Paucar et al.	Schiess et al.	Tzoulis et al.
Albumin	Low	Low	Normal	Normal	Low	Low	Low	Low
Cholesterol	Normal	Elevated	Elevated	Normal	Elevated	Elevated	Elevated	Elevated
IgE	Normal	Elevated	Normal	Elevated	Not available	Not available	Elevated	Not available
Alpha-fetoprotein	Elevated	Normal	Elevated	Elevated	Normal	Elevated	Normal	Normal

The occurrence of individuals homozygous or compound heterozygous for PNKP variants which are either homozygous or heterozygous for the MED25 variant indicates that the MED25 variant is a hitchhiker variant in linkage disequilibrium on the same haplotype with the PNKP variant p.Gln517del. Despite its relatively high population frequency (1067/274,696; gnomAD All), no additional CMT2 patients have been identified with variants in MED25. Only three patients were recently reported, but these patients had additional variants in other CMT-related genes, which could well explain their phenotype [3].

In our previous study, we demonstrated that in rats Med25 expression correlated with Pmp22 dosage and expression, which is interesting because Pmp22 is a gene involved in demyelinating peripheral neuropathies. We proposed that both Med25 and Pmp22 expression are regulated by neuron-Schwann cell interactions. In addition, we demonstrated that the p.Ala335Val variant in the mediator of transcription MED25 increases the activation of the target genes in the peripheral nervous system [2]. Considering this, and that both the p.Ala335Val mutation in MED25 and a mutation in codon 517 of PNKP have shown to modify the codified protein’s function [2, 12], and the late age of onset and mild affectation of the CR-P patients, it would be possible to hypothesize that the MED25 variant confers a protective effect in them. In order to test this, patients that only present the PKNP variant would be useful to compare them clinically with patients of the CR-P family.

The involvement of PNKP in neurodegenerative disorders was already reported by Poulton et al. (2013) who presented two cases affected with a progressive polyneuropathy with early onset, severe progressive cerebellar atrophy, microcephaly, mild epilepsy, and intellectual disability. A homozygous variant (c.1250_1266dup, p.Thr424GlyfsX48) in PNKP was identified in these cases [6]. This gene, mainly related so far with AOA4 and MCSZ, has also been found through exome analysis in an individual initially diagnosed with an axonal form of Charcot-Marie-Tooth disease. This individual was homozygous for the variant p.Thr408del, also identified in our study [14]. Therefore, this study confirms the role of PNKP mutations in peripheral polyneuropathies and add data associated to the variability of the PNKP-related phenotype, due to the late age of onset of the neuropathy in our PNPK mutated patients. In conclusion, we provide evidence that PNKP is the main gene related to CMT2B2 instead of MED25, and that it should be considered as a gene involved in an axonal peripheral neuropathy with late age onset, cerebellar atrophy, and with or without oculomotor apraxia.
